# A Comprehensive Analysis of the Agreement and Performance of Variant Annotation Programs in Equine Genomes

**DOI:** 10.3390/genes17060704

**Published:** 2026-06-18

**Authors:** Jillian L. Marlowe, Lauren Hughes, Eric Barrey, Tosso Leeb, Rebecca Bellone, Molly E. McCue, Sian Durward-Akhurst

**Affiliations:** 1 Department of Veterinary Clinical Sciences, University of Minnesota, St. Paul, MN 55455, USA; marlo072@umn.edu; 2 Department of Population Medicine, University of Minnesota, St. Paul, MN 55455, USAmccu0173@umn.edu (M.E.M.); 3 Genetique Animale et Biologie Intégrative UMR1313, AgroParisTech, Institut National de Recherche Pour l’Agriculture, l’Alimentation et l’Environnement, Université Paris-Saclay, 78350 Jouy-en-Josas, France; eric.barrey@inrae.fr; 4 Institute of Genetics, Vetsuisse Faculty, University of Bern, 3102 Bern, Switzerland; tosso.leeb@unibe.ch; 5 Veterinary Genetics Laboratory, School of Veterinary Medicine, University of California, Davis, CA 95616, USA; rbellone@ucdavis.edu; 6 Population Health and Reproduction, School of Veterinary Medicine, University of California, Davis, CA 95616, USA

**Keywords:** *Equus caballus*, horse, whole genome sequencing, methodology, tool validation

## Abstract

**Background/Objectives:** Advances in whole-genome sequencing (WGS) technology have led to the widespread adoption of WGS for investigating genetic diseases and traits in domestic animals. This has created a need for improved methods for prioritizing candidate causal variants. One way variants are prioritized is using variant annotators that predict variant effects based on their proximity to genomic features and effect on amino acid sequence. However, validation of variant annotators for domestic animal genomes is lacking. **Methods:** In this study, we calculated the agreement of three popular variant annotators, Ensembl Variant Effect Predictor (Ensembl-VEP), SnpEff, and ANNOVAR, across >58 million variants identified in 1065 horse genomes. **Results:** Comparisons showed that agreement across all three variant annotators was >90% when terminology was standardized. Terminology standardization was the most important factor affecting agreement, as agreement dropped to 0–67% when terminology was not standardized across variant annotators. Genomic context was also a major factor, as exonic, and specifically loss-of-function, variants showed lower agreement rates than intergenic variants. In addition to annotation agreement, differences in computational resource requirements were identified. ANNOVAR required ~28× more memory and ~1.5× more time than the next best tool. **Conclusions:** These results demonstrate that tool selection for annotating variants should not be based on a single metric; rather, a study’s needs and available computational resources should be considered when selecting the appropriate variant annotators(s) along with the standardization of terminology across annotators. These findings are a resource for guiding decisions on the use of variant annotators in domestic animals and suggest areas for improvement in the standardization of variant prioritization.

## 1. Introduction

Whole-genome sequencing is routinely used to investigate genetic diseases and other genetic traits of interest. However, accurately identifying the causal variant among the millions of genetic variants within a single genome remains challenging for multiple reasons [1]. Variant annotation, the process by which variants are interpreted based on their genomic location and predicted functional consequences, is an important step in distinguishing causal from neutral genetic variation. Many variant annotators also assign each variant an impact category based on its predicted consequence, which often serves as an additional filtering criterion when prioritizing variants for further investigation. Most variant annotators have been developed for and tested on human sequencing data. Yet despite their widespread use in domestic animals, these tools have not been critically evaluated or optimized specifically for domestic animal data.

An important question in determining optimal annotating procedures is whether using multiple variant annotators improves annotation sensitivity and specificity, particularly for clinically relevant variants such as those thought to cause loss-of-function (LOF). LOF variants that introduce premature stop codons, shift the reading frame, or alter splicing, may be of particular interest due to their high potential for impacting protein end products [2]. Sequencing and annotation errors can lead to identifying false positive LOF variants and many LOF variants are present only in certain transcripts for an affected gene [1]. Prior studies using human genomes have shown reasonable agreement of ~85–90% across all variants for the commonly used variant annotators Ensembl-VEP and ANNOVAR [3]. When focusing solely on exonic variants, human studies have shown only 86.5% agreement between the tools, with agreement dropping to 65% when considering only loss-of-function (LOF) variants [3].

In addition to the type of variant being evaluated, differences in variant annotator behavior, such as differences in variant classifications, the terminology used to describe classifications, and output prioritization decisions, make accurate comparisons difficult. This complicates variant annotator evaluation because nomenclature and transcript choice have an important impact on tool agreement [3,4,5,6,7]. It is therefore necessary to standardize classification terminology and definitions across variant annotators to ensure accurate comparisons. Standardization is facilitated by using Sequence Ontology (SO) terms, a controlled vocabulary with a specified set of relationships, initially developed by the Gene Ontology Consortium [8]. Efforts have been made to incorporate SO terminology into variant annotators; however, adherence to the standards varies between tools. A better understanding of how variant annotators perform and agree on domestic animal data and what factors impact agreement between tools will help researchers define appropriate filtering and prioritization steps to support best practices for variant discovery in these populations.

Here we evaluated the performance of three variant annotators that are often used in domestic animal genomic research: Ensembl Variant Effect Predictor (Ensembl-VEP) [9], SnpEff [10], and ANNOVAR [11]. We determined their agreement using equine WGS data from over 1000 horses as a model for domestic animal species. These tools are widely used and freely available and can be used to add annotation information directly to a Variant Call Format (VCF) file. We evaluated the agreement of annotations before and after terminology standardization to determine the best approach for cross-annotator standardizations. We also evaluated agreement among transcript-matched annotations as well as agreement among the most deleterious annotations made by each tool. This represents the first comparison of variant annotator performance on domestic animal data, in which the quality of genome annotation is less complete and less accurate than what is available for human-variant studies. 

## 2. Materials and Methods

### 2.1. Data Generation

A convenience sample of previously generated WGS data from 1068 sets of fastqs generated from 1067 horses, representing 48 distinct breeds and breed crosses, were used in this study (Table S1). The three most represented breeds were Thoroughbreds (n = 459), Standardbreds (n = 204), and Quarter Horses (n = 95). Two sets of fastqs were identified as duplicates of the same Thoroughbred horse during kinship analysis. Samples were collected from horses for use in several equine population genetics studies and disease discovery projects [12,13]. This data includes 75 publicly available genomes, 114 genomes shared by collaborators and 877 genomes generated by the University of Minnesota Equine Genetics and Genomics Laboratory (UMN EGGL). Sequencing data were generated primarily using Illumina sequencing technologies, with coverage depths ranging from 0.6× to 56× (median: 9.2×) (Figure S1). A cut-off of 75% mapping rate led to the removal of two poor-quality genomes, leaving 1065 horses for inclusion (Figure S2). 

**Figure 1:**
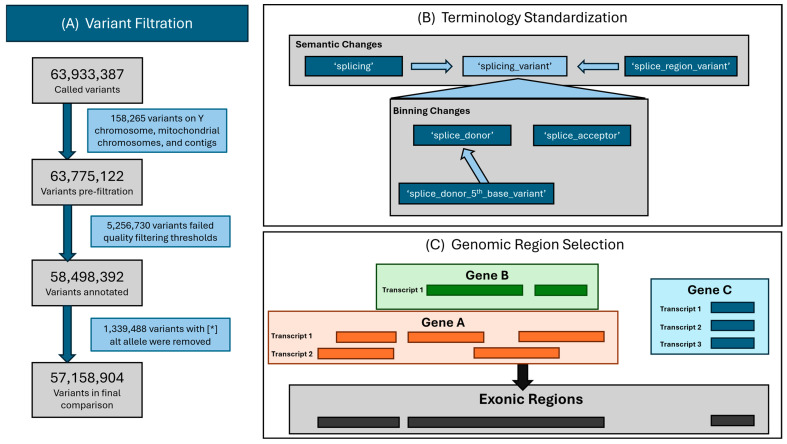
Methods for variant filtration, terminology standardization, and genomic region determination. (**A**) Variant processing steps showing the number of variants retained at each filtration step, including pre- and post-annotation steps. (**B**) Example of how terminology is standardized using possible terms relating to different kinds of splicing variants. Semantic changes solely standardize terms with the same definitions. Binning changes group very specific terms into broader classifications. (**C**) Visual demonstration of how overlapping exons present across transcripts are combined into continuous exonic regions.

Sequence processing was completed using the Whole Animal Genome Sequencing (WAGS) pipeline [14]. Briefly, sequence reads were mapped to the equine reference genome EquCab3 using the bwa-mem (v.0.7.17) algorithm [15]. Adapters and duplicates were marked by Picard (v.2.27.1) and GATK (v.4.2.3.0), respectively [16]. Base quality score recalibration was performed using known sites on the MNEc2M array [17]. Following alignment, GATK HaplotypeCaller was used for variant calling. gVCFs were then consolidated with GenomicsDBImport and joint genotyping performed on the entire dataset with GenotypeGVCFs [16]. A total of 63,933,386 variants were identified across all samples. Variants identified on the Y chromosome, mitochondrial chromosomes, and unmapped contigs were discarded due to insufficient genomic annotation in these regions of the horse genome. SNVs, insertions, and deletions (indels) were filtered using the Broad Institute’s minimum hard filtering threshold recommendations. For SNVs, filtering thresholds were QualitybyDepth < 2.0, Quality < 30.0, StrandOrientationRatio > 3.0, FisherStrand > 60.0, MappingQuality < 40.0, MappingQualityRankSum < −12.5, ReadPositionRankSum < −8.0. For indels, filtering thresholds were QualitybyDepth < 2, FisherStrand > 200, ReadPositionRankSum < −2, StrandOrientationRatio > 10 [18]. VQSR is not recommended for use in horses due to a lack of well-established truth sets for any variant type [19]. Alternate alleles notated with [*] by HaplotypeCaller were alleles representing a deletion that spans the position and were discarded due to incompatibility with annotation programs. Following filtration, 57,158,904 variants remained for annotation (Figure 1A).

### 2.2. Variant Annotation

A parallelized pipeline using Snakemake (v7.19.1) was developed to accelerate annotation of a large VCF (https://github.com/jlmarlo/BAndComp, accessed on 1 June 2026) [20]. Briefly, this pipeline splits the unannotated VCF into separate files for each chromosome. The VCF for each chromosome is then decomposed to split up multiallelic sites, followed by sequential annotation by Ensembl-VEP (v103), SnpEff (v. 5.2), and ANNOVAR (2020-06-08). For all three annotators, variants falling within 1000 base pairs (bp) of a gene boundary were classified as up- or downstream. Variants within 2 bp of a splice site were considered within a splice region for SnpEff and ANNOVAR. ANNOVAR and Ensembl-VEP do not allow the definition of a splice region to be changed. Ensembl-VEP considers splicing variants to occur up to three bases into an exon or up to eight bases into an intron. SnpEff splicing variant boundaries can be modified; however, it must be an even number on either side of the splice site, so it was standardized to 2 bp to match with ANNOVAR’s splicing variant definition.

This study used the Ensembl gene annotation files as they contain a larger number of genes and transcripts than other annotation sources, including RefSeq. The Ensembl v. 105 gene transfer format (GTF) for *Equus caballus* was used by Ensembl-VEP and ANNOVAR. The SnpEff database EquCab3v105 was used for annotation by SnpEff. A total of 29,462 genes were present in the GTF, with an average of 1.96 transcripts per gene. Within the database used by SnpEff, there were 30,371 genes, with an average of 1.95 transcripts per gene. By default, Ensembl-VEP and SnpEff generate annotations for all possible transcripts, while ANNOVAR only outputs the most deleterious annotation based on its internal precedence rules when outputting into VCF. Following annotation by all three variant annotators, the chromosome-specific VCFs were merged, and a tab-delimited file was generated containing location and annotation information for all three tools.

The complete annotation information generated by each variant annotator includes notations of the specific base pair and amino acid change, as well as a ‘classification’ (e.g., nonsynonymous variant) of the variant’s predicted functional effect. Ensembl-VEP and SnpEff also provide ‘impact’ predictions (e.g., high impact.) For the purposes of this study, we extracted the classification and impact prediction from each annotation. In total, Ensembl-VEP generated ~113 million (M) annotations, SnpEff ~109 M annotations, and ANNOVAR ~57 M annotations. 

### 2.3. Tool Comparisons

To account for differences in variant annotator terminology and methods, we used the following methods.

#### 2.3.1. Terminology Standardization

All three variant annotators classify variants using the variant’s physical location within or around a gene structure and its possible effects on the amino acid sequence of the corresponding transcript. However, these tools use different terminology to describe these classifications. SnpEff provides 58 variant classes and Ensembl-VEP provides 41 classes. ANNOVAR handles classification differently, with 9 primary classes, and an additional 11 sub-classifications for exonic variants for a total of 20 possible classifications (Table S2). To facilitate accurate comparisons, terminology was aligned in two ways. First, semantic changes were performed. Semantic changes were made when variant annotators used different terms for variant classifications that share identical or nearly identical definitions. For example, ANNOVAR uses the term ‘upstream’ to describe a variant that lies within 1000 bp of the 5′ end of a gene, while Ensembl-VEP and SnpEff use ‘upstream_gene_variant’. Semantic changes were made so that classifications conformed to the terms from the Sequence Ontology (SO) Browser [8]. In the previous example, ANNOVAR’s term would be changed to ‘upstream_gene_variant’, which is the SO term. 

Secondly, in cases where a more specific classification fits into a broader parent SO category, ‘binning’ to the level of the broader term was performed to facilitate agreement. For example, SnpEff and Ensembl-VEP provide classifications for ‘splice_acceptor’ and ‘splice_donor’ variants, but ANNOVAR simply classifies any variant occurring within a splice region as ‘splicing’. Since splice acceptor and splice donor variants are specific types of splicing variants, these classifications were binned under the SO term ‘splicing_variant’ (Figure 1B). In cases where a classification term used by one of the variant annotators is unique and does not readily fit within a parent SO term, the original terminology was left unchanged (Table S3).

#### 2.3.2. Matched-Transcript and Highest-Precedence Comparisons

There are also differences in how each variant annotator handles a variant that is present in multiple transcripts of the same gene. Ensembl-VEP and SnpEff provide annotations for every possible transcript containing the given variant. In contrast, when outputting in VCF, ANNOVAR outputs only the annotation that results in the most deleterious classification based on internal precedence rules and does not provide annotations for the remaining isoforms. Ensembl-VEP and SnpEff may also give multiple classifications for a single transcript. This often occurs when the ‘best’ classification is ambiguous. For example, an insertion that begins at a splice site and causes a frameshift can have multiple effects and may be classified as both a splicing variant and a frameshift variant (i.e., ‘*splice_variant&frameshift_variant’*). 

Ensembl-VEP and SnpEff also provide impact predictions, which are broad estimates of how significantly a variant may affect protein function. Possible impacts range from ‘MODIFIER’, which indicates a variant that is unlikely to have an impact on protein function, to ‘HIGH’, which indicates a variant that may cause complete loss of function. ANNOVAR does not provide impact predictions.

To account for these differences in operation, we developed two complementary methods of comparing annotations. For *Matched-Transcript Comparisons*, annotations from Ensembl-VEP and SnpEff were matched by transcript ID for each variant in the dataset and corresponding classifications and impacts were compared. The classification produced by ANNOVAR was compared to all classifications generated by the other two tools regardless of transcript ID. In cases where a tool or multiple tools gave a list of possible classifications for a single transcript, the overlap of variant annotators among the lists was first determined. If the lists matched fully, this was considered agreement. If there was no overlap between the lists, it was considered a disagreement. If the lists overlapped but contained unique items in one or both lists, it was considered a partial match. In addition to variant classifications, impact predictions from Ensembl-VEP and SnpEff were also compared for each transcript.

For *Highest-Precedence Comparisons*, comparisons were made only for the most deleterious classification predicted by each variant annotator for a variant based on the precedence lists of each tool. Precedence lists detailing the order of severity of deleterious variants were obtained from the online documentation for each tool (Table S4). For each variant, the highest-precedence classification for each tool was determined, and these classifications (and impacts in the case of Ensembl-VEP and SnpEff) were compared. The number of times all three variant annotators disagreed was also determined.

### 2.4. Genomic Context

To evaluate if variant annotator agreement varies by genomic context, comparisons were performed on variants in intergenic, genic, and exonic regions using the matched-transcript comparison method described above. The completely annotated VCF was divided into separate intergenic, genic, and exonic VCFs. Genic regions were defined as 1000 base pairs (bps) from the gene’s transcriptional start and stop positions in the E. caballus v105 GTF. Loci outside of these regions were considered intergenic. Insertions or deletions that overlapped the boundaries of a genic region were considered genic even if they originated outside of the genic region. To determine exonic regions, records labeled ‘exon’ in the GTF were identified, and 2 bp was added to the start and end positions to account for splice regions. This was done on a transcript-by-transcript basis. Exons that overlap each other were combined to make continuous exonic regions that may contain multiple individual exons across transcripts or genes (Figure 1C). Variants identified within any exonic region, including insertions and deletions that cross exon boundaries, made up the exonic dataset. Intergenic, genic, and exonic VCFs were converted to tab-separated files, and comparisons were performed as described above. In addition, the agreement among LOF variants was calculated. LOF variants were defined as those likely to result in loss or serious impairment of gene product function. This category of variants included predicted gene fusions, exon losses, frameshifts, splicing variants, start and stop losses, and stop gains. If a variant was predicted to have a LOF classification by at least one variant annotator it was considered a LOF variant. LOF variants were compared using the highest-precedence comparison method.

### 2.5. Identification of Discordant Classifications

To identify classifications that were most likely to contribute to disagreements between programs, we quantified disagreement in two complementary ways based on the highest-precedence comparison method. First, we summarized observed disagreements by calculating the percentage that each specific conflict (e.g., upstream vs. downstream) made up of all disagreements that occurred between two tools. However, this approach is inherently influenced by the frequency of each classification across the genome (i.e., disagreements involving highly abundant classification, such as upstream or intronic variants, are more likely to have high numbers of occurrence simply due to the prevalence of those classifications and therefore make up a large percentage of the total disagreements). Therefore, we also assessed disagreement on a per-classification basis by calculating the percentage of each classification that disagrees with the output of the other tools (e.g., 80% of stop-gain classifications annotated by Ensembl-VEP received a different classification from SnpEff). Annotation classifications that had > 10% disagreement rate in a tool comparison were considered major contributors to either method.

### 2.6. Computational Performance Evaluation and Statistical Testing

To evaluate differences in the computational resources used by each variant annotator, a modified version of the annotation pipeline was created. All three tools were run independently on identical VCFs that contained no annotation information. For each variant annotator, five repetitions were performed on each chromosome, and runtime and memory usage were recorded. Each repetition was allocated a maximum of 200 gigabytes of memory. These replicates yielded a total of 160 measurements (32 chromosomes x 5 replicates) per tool. To estimate the computational resources required for any application, the average runtime and memory used per megabase and per 10,000 variants were calculated. 

The distribution of the data within each group for each measure was assessed for normality using the Shapiro–Wilk test and for homogeneity of variances using Levene’s test. Data did not meet the assumptions of normality and homoscedasticity, and nonparametric tests were thus utilized for statistical testing. Differences among the three variant annotators were evaluated using a Kruskal–Wallis rank-sum test. Post hoc testing was performed using Dunn’s test with a Bonferroni adjustment for multiple comparisons. Pearson’s correlation coefficient was calculated to assess the relationship between the number of variants evaluated and the time and memory required to complete annotation.

## 3. Results

### 3.1. Variant Annotation

Of the 57,158,904 variants analyzed, the most common variant classifications were non-coding, such as intergenic, intronic, upstream, and downstream (Table 1). Exonic variants accounted for 3.4% of the annotated variants and 0.5% were predicted to be LOF.

### 3.2. Tool Agreement

#### 3.2.1. Terminology Effects

**Figure 2:**
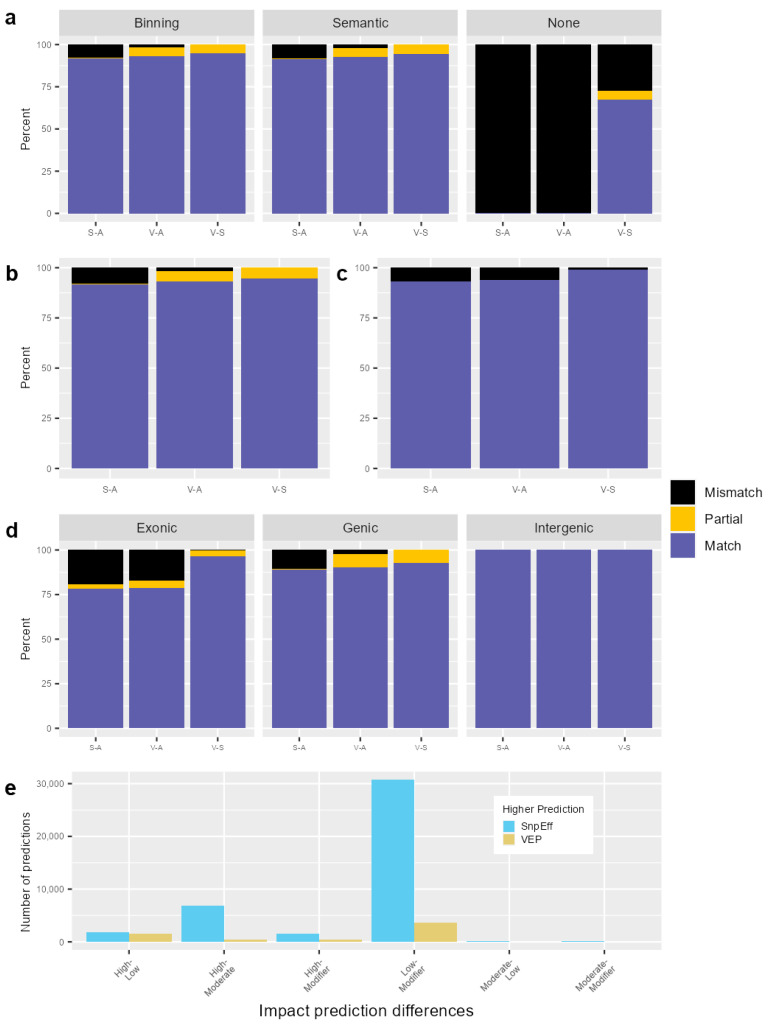
Agreement rates of variant classifications and impact predictions between variant annotators. (**a**–**d**) Classification agreement between SnpEff (S), VEP (V), and ANNOVAR (A), shown as the percentage of matching (purple), partially matching (yellow), or mismatching (black) variants. (**a**) Effect of terminology standardization on agreement rates, showing that semantic standardization results in the largest increase in agreement in all tool comparisons. (**b**) General agreement of pairwise tool comparisons using the matched-transcript approach (**c**) General agreement of tools using the highest-precedence comparison method (**d**) Changes in tool agreement depending on genomic context, showing that agreement decreases in genic and exonic regions. (**e**) Count and direction of impact prediction disagreements between SnpEff (blue) and VEP (beige). The variant annotator that predicts the more deleterious impact is indicated, showing that SnpEff predicts a higher impact than VEP.

Standardization of terminology played a large role in the agreement of all variant annotators. Prior to standardization of classification terms, agreement between tools was low. Ensembl-VEP and SnpEff had an overall agreement of 67.4%, and ANNOVAR had 0.1% agreement with the other tools due to non-overlapping terminology (Figure 2c). In total, 86,739,475 semantic changes were made, and 4,096,533 classifications were binned into broader classifications (Table S3). Ensembl-VEP required the fewest changes to make standardized comparisons, with only 1.4% of classifications changed; SnpEff required 29.5% and ANNOVAR required 100% of classifications to be standardized. For all three variant annotators > 90% of all changes were semantic changes (biologically identical classifications) while <10% of changes were binning changes (simplified, less specific classifications). The largest increase in agreement occurred following semantic changes. Ensembl-VEP and SnpEff agreement increased by ~30% (to 94.6%), and comparisons with ANNOVAR increased by over 90% (Ensembl-VEP: 92.6%, SnpEff: 91.5%). When binning changes were included, agreement between tools increased by less than 1% in all cases (Ensembl-VEP–SnpEff: 94.7%, Ensembl-VEP–ANNOVAR: 93.0%, SnpEff–ANNOVAR: 91.9%) (Figure 2c). 

#### 3.2.2. Matched-Transcript Comparison

In total, 323,410,142 matched-transcript comparisons were made between the three variant annotators. Agreement was above 90% across all tools. Ensembl-VEP and SnpEff had the highest level of agreement (94.7%), followed by Ensembl-VEP and ANNOVAR (93%), while SnpEff and ANNOVAR had the lowest agreement (91.9%). For comparisons involving Ensembl-VEP, classifications that did not fully agree were likely to at least partially agree (SnpEff: 5.3%, ANNOVAR: 5.5%), with a very low rate of classifications in complete disagreement (SnpEff: 0.01.%, ANNOVAR: 1.6%). The opposite was true for comparisons between SnpEff and ANNOVAR, where classifications were more likely to fully disagree than partially disagree (partial: 0.3%, disagree: 7.8%) (Figure 2b). Impact predictions made by SnpEff and Ensembl-VEP agreed 99.96% of the time, with only 47,094 (0.04%) of predictions disagreeing. These disagreements were for 23,314 (0.0004%) of the > 57 million variants annotated due to variants affecting multiple transcripts. When impact prediction mismatches occurred, 88.6% were differences between adjacent impact categories—for example, moderate to high (15.4%) or modifier to low (72.9%)—with only 11.4% separated by more than one impact category. SnpEff tended to assign a higher impact more often than Ensembl-VEP (Figure 2e).

#### 3.2.3. Highest-Precedence Comparison

A total of 171,476,712 highest-precedence comparisons were made, corresponding to one comparison per variant per tool pair. Agreement of variant annotators followed the previous pattern, with SnpEff and Ensembl-VEP being the most concordant (98.9%), Ensembl-VEP and ANNOVAR in second place (93.9%), and SnpEff and ANNOVAR agreeing the least (93.0%). Because only the highest-precedence classifications were compared, partial matches were not possible for these comparisons. The disagreement rate between tools increased with the highest-precedence comparison compared to the matched-transcript comparison (Ensembl-VEP–SnpEff: 1.1%; Ensembl-VEP–ANNOVAR: 6.1%; SnpEff–ANNOVAR: 7.0%) (Figure 2b). There were 129,919 variants (0.23%) out of 57 million that resulted in a triple disagreement, with each variant annotator assigning a different classification.

#### 3.2.4. Genomic Context

A total of 29,873,679 intergenic, 28,624,713 genic, and 1,943,682 exonic variants were identified. There was no overlap between the intergenic VCF and the genic VCF, while there was 100% overlap of exonic variants with the genic VCF, confirming the correct separation of variants. Agreement across all three variant annotators was 100% in intergenic regions, as the only classification in these regions was ‘intergenic_variant’. Agreement was lowest for exonic region classifications when comparing ANNOVAR and the other two tools (VEP: 78.8%, SnpEff: 78.1%). Agreement among Ensembl-VEP and SnpEff was 96.4% in exonic regions and lowest (92.7%) in the broader genic regions (Figure 2d).

LOF variants exhibited the lowest agreement rate. There were 299,979 variants classified as loss-of-function by at least one variant annotator. For LOF variants, agreement between Ensembl-VEP and SnpEff decreases to 92.1% agreement, and agreement with ANNOVAR drops to 71.4% for Ensembl-VEP and 68.7% for SnpEff.

### 3.3. Identification of Discordant Classifications

Certain classifications accounted for the most disagreements between variant annotators when classifications were compared using the highest precedence. The most common disagreements between Ensembl-VEP and SnpEff occurred when Ensembl-VEP classified a variant as ‘intron_variant,’ and SnpEff classified the variant either as ‘downstream_gene_variant’ (44.1% of disagreements) or ‘upstream_gene_variant’ (39.2% of disagreements). For comparisons between Ensembl-VEP or SnpEff and ANNOVAR, nearly all disagreements occurred when Ensembl-VEP or SnpEff classified a variant as ‘intron_variant’ and ANNOVAR classified it as ‘non_coding_transcript_variant’ (Ensembl-VEP: 97.5%, SnpEff: 83.4%). The classifications in which all three tools disagreed reflected the disagreements between the paired comparisons. Variants that Ensembl-VEP classified as ‘intron_variant’, SnpEff tended to classify as either ‘downstream_gene_variant’ or ‘upstream_gene_variant’, and ANNOVAR tended to classify as ‘non_coding_transcript_variant’ making up 98.7% of variants where all three tools gave different classifications.

Loss-of-function classifications with high rates of disagreement are highly variable depending on which variant annotators are being compared. The classification ‘start_loss’, made by Ensembl-VEP, had high rates of disagreement in comparison with SnpEff. Comparing Ensembl-VEP and ANNOVAR, the ‘splicing_variant’ classifications made by Ensembl-VEP and ‘stop_gained’ and ‘stop_lost’ classifications from ANNOVAR had high rates of disagreement. When SnpEff is compared to ANNOVAR, only ‘splicing_variant’ classifications made by SnpEff showed high rates of disagreement, and ANNOVAR classifications of ‘stop_gain’ and ‘stop_loss’ were frequently in disagreement with SnpEff (Table 2).

### 3.4. Computational Performance Comparison

**Figure 3:**
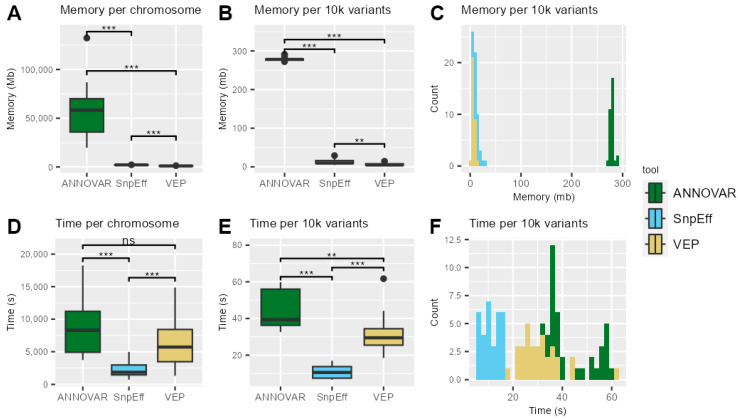
Memory usage and runtime performance of variant annotators. (**A**–**C**) Memory usage comparisons. (**A**) Average memory usage per chromosome. (**B**) Average memory usage per 10k variants calculated for each chromosome. (**C**) Distribution of memory usage per 10k variants (**D**–**F**) Runtime comparisons. (**D**) Average runtime taken per chromosome (**E**) Average runtime per 10k variants calculated per chromosome (**F**) Distribution of runtime per 10k variants. Boxplots show medians and interquartile ranges, with points representing outlying chromosomes. Statistical significance between tools is indicated by brackets (ns—not significant; ** *p* < 0.01; *** *p* < 0.001). Colors denote variant annotator: ANNOVAR (green), SnpEff (blue), and VEP (yellow).

There were statistically significant differences in the memory required and the time needed to complete annotations among the three variant annotators (Memory: *H*(2) = 84.45, *p* < 2.2 × 10^−16^; Time: *H*(2) = 55.95, *p* = 7.09 × 10^−13^). Memory usage was significantly different for all pairwise tool comparisons (Ensembl-VEP-SnpEff: *p* = 1.3 × 10^−5^, Ensembl-VEP-ANNOVAR: *p* = 1.18 × 10^−19^, SnpEff-ANNOVAR: *p* = 1.3 × 10^−5^). For time requirements, SnpEff was significantly different from VEP (*p* = 8.54 × 10^−7^) and ANNOVAR (*p* = 1.01 × 10^−12^). ANNOVAR had the highest median memory requirements (55,401 Mb [IQR: 34,170 Mb]) and the longest runtime (8909 s [IQR: 6290 s]). Ensembl-VEP was the most efficient with memory usage (memory: 1029 Mb [IQR: 38.8 Mb]; time: 6241 s [IQR: 4955 s]) while SnpEff was the most time-efficient (memory: 2099 Mb [IQR: 37.5 Mb]; time: 2169 s [IQR: 1520 s]). The correlation between the number of variants annotated and the time and memory required by SnpEff (time: R^2^ = 0.83 [CI: 0.67–0.91]; memory: R^2^ = 0.90 [CI: 0.80–0.95]) and ANNOVAR (time: R^2^ = 0.86 [CI: 0.73–0.93]; memory: R^2^ = 1.00 [CI: 0.99–0.99]) was high. For Ensembl-VEP, memory was not significantly correlated (R^2^ = 0.09 [CI: -0.27–0.42]), but time was (time: R^2^ = 0.88 [CI: 0.77–0.94]). Based on these correlations, the memory and time required per 10,000 variants for each variant annotator were calculated. The median memory required to process 10,000 variants was 4.83 Mb (Ensembl-VEP [IQR: 3.66 Mb]), 9.90 Mb (SnpEff [IQR: 7.53 Mb]), and 278 Mb (ANNOVAR [IQR: 2.71 Mb]). The median amount of time required per 10k variants was 10.6 (SnpEff [IQR: 6.21 s]), 29.5 (Ensembl-VEP [IQR: 8.96 s]), and 39.3 (ANNOVAR [IQR: 19.7 s]) seconds (Figure 3).

## 4. Discussion

This study is the first large-scale evaluation of agreement and computational performance among three commonly used variant annotators (Ensembl-VEP, SnpEff, and ANNOVAR) in a non-human species, the domestic horse. By comparing these tools across ~57 million variants from equine genomes, we show that overall annotation agreement is high between all tool pairs (>90%). Tool concordance is affected by terminology, genomic context, and differences in how tools predict or prioritize variant functional effects. These findings provide important insight for researchers working in non-human species, where variant annotator choice is often guided by practices established in human genomics but, until now, not validated in other species. 

Across the genome, pairwise agreement between variant annotators exceeded 90% using both the transcript-matched and highest-precedence approaches when terminology was standardized. Overall, SnpEff and Ensembl-VEP exhibited the highest agreement, whereas SnpEff and ANNOVAR agreed least, although the total numerical disagreements were relatively small. The highest-precedence approach was designed to mirror ANNOVAR’s annotation-prioritization logic. However, applying this approach did not improve concordance between ANNOVAR and the other tools. In most cases, disagreement increased because partial matches in transcript comparisons often resulted in complete disagreements under the precedence approach. These patterns suggest that although all variant annotators provide broadly similar information, there are actionable differences in tool selection. In combination, these results are consistent with earlier human-based studies that reported high genome-wide concordance between combinations of popular variant annotators ranging between 85 and 93.3% [3,4,5,7].

The impact predictions (HIGH, MODERATE, etc.) used by Ensembl-VEP and SnpEff were more concordant (99.96%) than their underlying classifications (94.7%) (nonsynonymous, frameshift, etc.). This is likely because they are broader predictions compared to the more specific variant classifications. Many published studies in the horse have prioritized variants for further evaluation based on impact categories [12,13,21]. These data support this approach over relying specifically on underlying classifications. Our findings suggest that functional prioritization pipelines that use impact predictions would be less sensitive to discrepancies among variant annotators. When disagreements did occur, they most commonly involved adjacent impact levels rather than large differences in predicted impact. Notably, SnpEff more frequently assigned higher impact levels than Ensembl-VEP, suggesting consistent differences in how conservative each tool may be in predicting impact. These patterns indicate that while classification disagreements between these two variant annotators are important to be aware of, practical annotation pipelines that use impact predictions may circumvent most such disagreements. 

The largest source of discordance comes from differences in classification terminology. Without standardizing terminology, Ensembl-VEP and SnpEff shared only moderate agreement in variant classification (~67%), while ANNOVAR showed ~0% agreement with either tool. ANNOVAR does not use SO terminology. When we standardized the terms, agreement levels increased sharply across all variant annotators, exceeding 90% in every pair-wise comparison made. This demonstrated that most disagreements between tools resulted from terminology rather than differences in variant interpretation. Although previous papers have advocated adopting a standardized ontology, to our knowledge, none has quantified how dramatically variant annotator agreement depends on the consistency of terminology. These findings highlight a major practical barrier for researchers comparing annotation results across tools or, most importantly, comparing findings to previous studies. When different variant annotators are used across studies, reconciling annotation terminology will be needed before meaningful interpretations across tools are possible. 

Interestingly, binning specific variant classifications (i.e., ‘splice_acceptor_variant’) into broader classifications (i.e., ‘splicing_variant’) did not produce meaningful gains in agreement (less than 1% improvement across all comparisons). This is likely because only a small number of variants fell within these classifications (4.6%). This suggests that, for some annotations, broadening classifications loses useful specificity without improving tool agreement. Tools that rely on physical location for annotation information are not necessarily optimized for these detailed effect predictions. This may be particularly true for domestic animal species, which tend to have less comprehensive genome annotations than humans. For these species splice sites or open reading frames are likely to be predicted rather than experimentally determined and validated. These observations reinforce the importance of adopting standard terminology when using multiple variant annotators to prioritize variants for further evaluation. 

Agreement between variant annotators varies substantially across genomic regions. Variants within intergenic regions showed the highest agreement, likely because their classification is straightforward. In contrast, genic and exonic regions, areas where functional interpretation is most utilized in genomic research, showed lower agreement. This echoes findings from previous human studies, in which agreement across variant annotators decreased for functionally relevant variants [3]. This likely relates to the uncertainty regarding the predicted gene annotations, especially when multiple isoforms are considered. In horses, the difference between intergenic and genic regions may be amplified by missing regulatory annotation, meaning variants outside annotated regions are uniformly labeled as ‘intergenic’, reducing opportunities for disagreement. The reduced agreement within known functionally relevant regions is important because these are the variants researchers typically prioritize. Moreover, variants that were predicted to be LOF by at least one variant annotator showed the lowest agreement (68–92%), consistent with prior reports that LOF annotation is the most inconsistent annotation type [3]. This suggests that genomic areas of high biological interest are those with the lowest between-tool agreement. Therefore, using two or more variant annotators may increase sensitivity in identifying variants to prioritize within genic regions by increasing the number of variants that receive a deleterious prediction by at least one tool. This decreases the likelihood of missing biologically relevant variants if only one tool is used.

We examined the classifications with the highest level of disagreement and found that most disagreements between Ensembl-VEP and SnpEff involved variants classified as intronic by Ensembl-VEP and upstream or downstream by SnpEff. This difference is driven almost exclusively by precedent rather than biology, because VEP considers intronic variants more deleterious than upstream or downstream variants whereas SnpEff does the opposite. These categories share equivalent predicted impact scores, but according to the tool documentation, the ranked precedence of these categories differs across tools, resulting in different transcripts being compared. For ANNOVAR, disagreement frequently arose from its tendency to classify variants as ‘non-coding variants,’ possibly because the program treats transcripts with incomplete open reading frames as non-coding transcripts. This behavior could have substantive implications for organisms with less complete genome annotation than the horse, where there may be a lack of well-defined coding regions. 

Beyond annotation agreement, we also evaluated computational performance, an aspect that has been largely absent from previous studies comparing variant annotators. Runtime and memory requirements differed substantially between the tools. ANNOVAR required the greatest memory and longest runtime, likely reflecting its processing pipeline and the additional resources necessary to convert its typical text file outputs into VCFs. SnpEff was fastest while Ensembl-VEP used the least memory. Because these comparisons were conducted under high-volume workload conditions on a high-performance computing system, local performance or use for smaller datasets may vary. We provide estimates for computational performance in high-performance computing environments using parallelization and with high memory allocations. True program performance may differ with changes in hardware or parallelization strategy. These findings provide empirical guidance for users whose tool selection is constrained by computational availability or data-throughput requirements.

### Limitations

Genome annotation for the horse has primarily been carried out using in silico predictions, with comparatively little information provided by gene models built from transcriptome data. This restricts the information available to variant annotators to make predictions. Future versions with more functional validation and additional transcript-level data would increase the accuracy of variant annotations. This study only assessed annotation agreement between tools, not the accuracy of the variant annotators for predicting the true functional effect of a variant. Predictions made by variant annotators may not be biologically accurate. Annotations made for transcripts that are not expressed in the tissues or cells of interest, or cases where annotations made for transcripts that are inaccurate themselves, are only some of the ways that this may happen. Unfortunately, there are very few variants with functional consequences that have been experimentally validated in horses. This makes it impossible to calculate and compare the prediction accuracy of the variant annotators in horses. This limits the comparisons that can be made. Additionally, this work used only the Ensembl gene set for comparisons. In human data, there is considerably more discordance between annotations made with Ensembl and RefSeq gene definition files [3]. The same is likely true for the horse and other domestic animals. Future work should explore annotation accuracy and agreement as genome annotation improves by adding transcriptomic and functional annotation data. This will allow for the assessment of the impact of regulatory features on annotation. Efforts should also be made towards the development and implementation of a standardized ontology that will enable cross-study comparisons without manual intervention.

## 5. Conclusions

We found that, similar to work in humans, agreement between commonly used variant annotators Ensembl-VEP, SnpEff, and ANNOVAR is high when compared using the equine genome assembly and annotations. The most important factor in tool agreement is the standardization of terminology though genomic context also impacts agreement rates. Output prioritization rules further impact agreement. In addition, we found significant differences in the time and memory usage of all three variant annotators. Taken together, these findings suggest that the appropriate tool selection is unlikely to be based solely on annotation agreement or computational performance. Instead, tool selection will depend on multiple interacting considerations, including expected accuracy, sensitivity to genome annotation quality, desired output granularity, compatibility with downstream software, and available runtime and memory. For many applications, especially those involving functional variant interpretation, using multiple variant annotators and standardizing outputs may increase the reliability of annotations. Researchers using variant annotation programs should explicitly report the specific annotation tools and tool version they use to ensure the transparency, reproducibility, and appropriate interpretation of results.

## Figures and Tables

**Table 1:** Top five most common variant classifications per annotator prior to terminology standardizations (percent of all classifications made by tool).

VEP	SnpEff	ANNOVAR
intron_variant (62.6%)	intron_variant (65.2%)	intergenic (51.1%)
intergenic_variant (25.8%)	intergenic_region (27.9%)	intronic (37.5%)
non_coding_transcript_variant (4.9%)	synonymous_variant (1.7%)	ncRNA_intronic (6.0%)
synonymous_variant (1.6%)	downstream_gene_variant (1.5%)	synonymous_SNV (1.2%)
downstream_gene_variant (1.4%)	upstream_gene_variant (1.3%)	downstream (1.1%)

**Table 2:** Proportions of times that loss-of-function classifications disagree within a given tool comparison.

	Ensembl-VEP-SnpEff		Ensembl-VEP-ANNOVAR		SnpEff-ANNOVAR	
LOF Classification	Ensembl-VEP	SnpEff	Ensembl-VEP	ANNOVAR	SnpEff	ANNOVAR
stop_gained	8.6%	0.11%	3.5%	28.2%	0.86%	38.0%
start_lost	17%	2.6%	NA	NA	NA	NA
frameshift	1.1%	1.7%	1.3%	3.6%	1.7%	3.4%
stop_lost	2.2%	1.5%	4.1%	38.3%	3.0%	38.1%
splicing_variant	2.2%	3.9%	24.1%	1.1%	23.4%	2.2%

## Data Availability

For all genomes used in this study, unfiltered variant information was deposited in the European Variant Archive at accession number PRJEB114396.

## Supplementary Materials

The following supporting information can be downloaded at: https://www.mdpi.com/article/10.3390/genes17060704/s1, Table S1: Breed Counts; Figure S1: Distribution of sample depth of coverage; Figure S2: Distribution of sample mapping percentage; Table S2: Classification counts prior to terminology standardization; Table S3: Counts of all semantic and binning changes made during terminology standardization; Table S4: Precedence Rankings for Annotation Tool Classifications.

## Abbreviations

The following abbreviations are used in this manuscript:

WGS	Whole Genome Sequencing
VEP	Variant Effect Predictor
LOF	Loss-of-function
SO	Sequence Ontology
VCF	Variant call format
VQSR	Variant Quality Score Recalibration
GTF	Gene Transfer Format
