# Supplementary material for: A Comprehensive Analysis of the Agreement and Performance of Variant Annotation Programs in Equine Genomes
Source: Genes (Basel). 2026 Jun 18;17(6):704. doi: 10.3390/genes17060704 (PMC13299172; doi:10.3390/genes17060704)
Supplement: Supplementary file 1 [file genes-17-00704-s001.zip › genes-4310280-supplementary.pdf]

Table S1. Breed counts.

Breed	Count	Breed	Count
Appaloosa	1	Mongolian Horse	4
Arabian	37	Morgan	21
Baden Wuerttemberg	1	Norwegian Fjord	1
Bavarian Warmblood	1	Oldenberg	2
Belgian	20	Paint	3
Clydesdale	19	Palomino	2
Coldblood	4	Percheron	3
Connemara	4	Pony of America	1
Curly Trotter	2	Pony	2
Duelmener	1	Quarter Horse	95
Franches-Montagnes	30	Saddle Trotter	2
French Trotter	10	Saxon-Thuringian Heavy Warmblood	1
Friesian	2	Shetland	29
Haflinger	7	Sorraia	2
Hanoverian	4	Sports Horse	2
Holsteiner	3	Standardbred	204
Icelandic	18	Swiss Warmblood	1
Italian Trotter	1	Tennessee Walking Horse	4
Jeju Pony	4	Thoroughbred	459
KWPN	1	Trakehner	2
Lipizzaner	4	Unknown	14
Mangalarga Marchador	4	Warmblood	6
Miniature Horse	4	Welsh Pony	20
Missouri Fox Trotter	1	Westphalian	2

*Crossbreeds are represented by the owner-reported breed.

Figure S1. Distribution of sample depth of coverage.

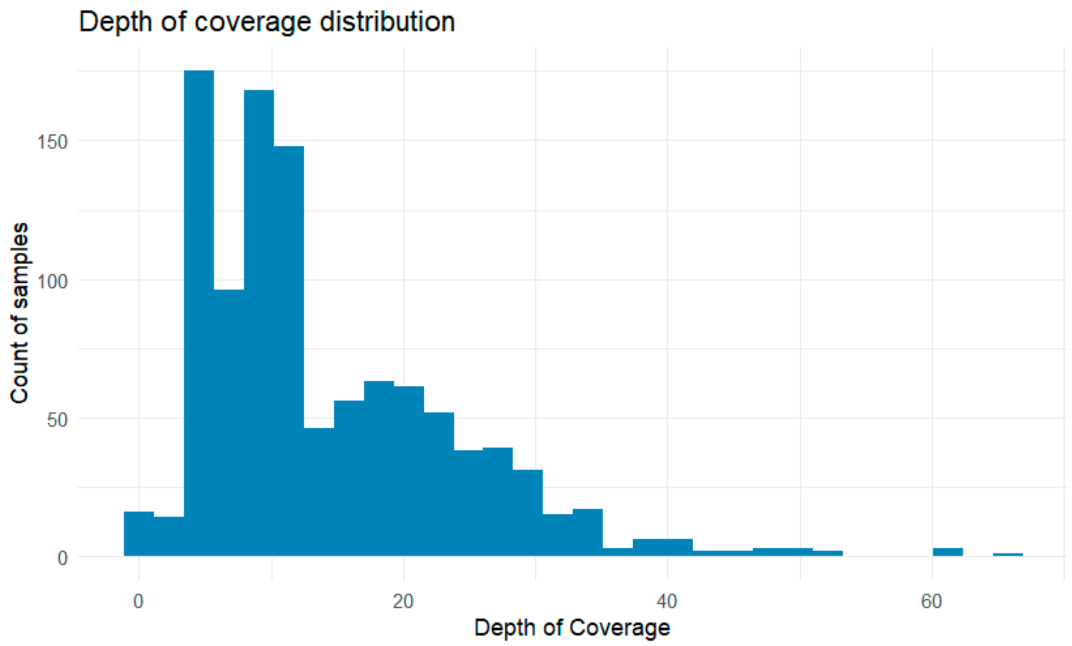

Figure S2. Distribution of sample mapping percentage.

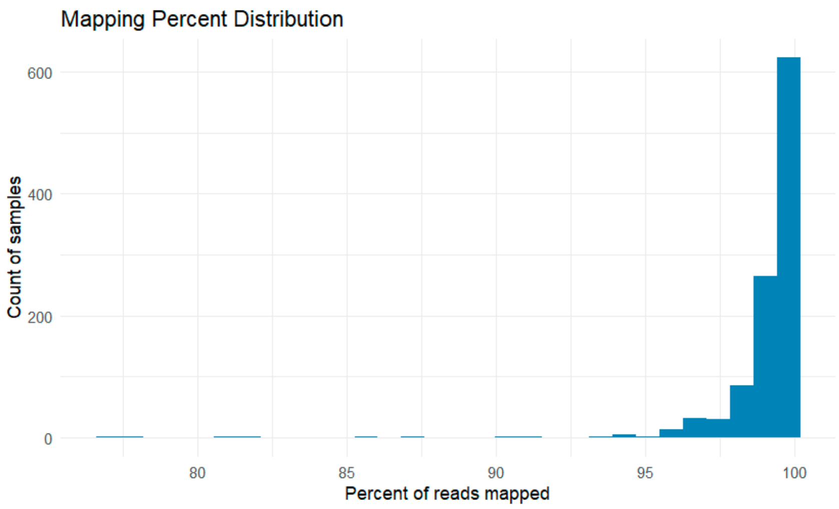

Table S2. Classification counts prior to terminology standardization.

VEP Classes		SnpEff Classes		ANNOVAR Classes	
Classification	Counts	Classification	Counts	Classification	Counts
intron_variant	70,962,196	intron_variant	71,026,323	intergenic	29,197,844
intergenic_variant	29,196,742	intergenic_region	30,403,316	intronic	21,439,491
non_coding_transcript_variant	5,580,986	synonymous_variant	1,813,385	ncRNA_intronic	3,410,079
synonymous_variant	1,813,288	downstream_gene_variant	1,603,709	synonymous_SNV	704,339
downstream_gene_variant	1,603,191	upstream_gene_variant	1,401,435	downstream	649,889
upstream_gene_variant	1,400,731	missense_variant	953,746	upstream	579,004
missense_variant	953,503	frameshift_variant	424,915	nonsynonymous_SNV	417,365
frameshift_variant	427,122	3_prime_UTR_variant	418,092	ncRNA_exonic	252,704
3_prime_UTR_variant	418,363	non_coding_transcript_exon_variant	308,368	UTR3	202,677
non_coding_transcript_exon_variant	308,479	splice_region_variant	259,357	frameshift_deletion	151,690
splice_region_variant	241,558	5_prime_UTR_variant	170,251	UTR5	75,245
5_prime_UTR_variant	170,770	splice_donor_variant	54,834	splicing	25,407
inframe_deletion	43,373	splice_acceptor_variant	42,495	frameshift_insertion	23,817
splice_donor_variant	41,333	disruptive_inframe_deletion	30,315	nonframeshift_deletion	20,155
splice_acceptor_variant	32,152	stop_gained	27,485	stopgain	18,004
stop_gained	31,081	5_prime_UTR_premature_start_codon_gain_variant	19,889	nonframeshift_insertion	9,809
inframe_insertion	21,134	conservative_inframe_deletion	16,348	ncRNA_splicing	1,434
coding_sequence_variant	17,570	conservative_inframe_insertion	8,813	stoploss	994
protein_altering_variant	3,007	disruptive_inframe_insertion	8,424	exonic	925
stop_lost	1,886	intragenic_variant	5,234	ncRNA_UTR5	7
start_lost	1,845	stop_lost	2,083		

stop_retained_variant	1,033	start_lost	1,588		
start_retained_variant	35	stop_retained_variant	1,008		
transcript_ablation	10	non_coding_transcript_variant	729		
		initiator_codon_variant	241		
		exon_loss_variant	223		
		start_retained_variant	102		
		bidirectional_gene_fusion	44		
		gene_fusion	24		
		5_prime_UTR_truncation&exon_loss_variant	17		
		transcript_ablation	10		
		3_prime_UTR_truncation&exon_loss_variant	4		
Total	113,271,388	Total	109,002,807	Total	57,180,879

Table S3. Counts of all semantic and binning changes made during terminology standardization.

VEP		
Original	Altered	Count
splice_region_variant	splicing_variant	241,558
missense_variant	nonsynonymous_variant	953,503
non_coding_transcript_exon_variant	non_coding_transcript_variant	308,479
splice_acceptor_variant	splicing_variant	32,152
splice_donor_variant	splicing_variant	41,333
stop_retained_variant	synonymous_variant	1,033
start_retained_variant	synonymous_variant	35

SnpEff		
intergenic_region	intergenic_variant	30,403,316
missense_variant	nonsynonymous_variant	953,746
splice_region_variant	splicing_variant	259,357
disruptive_inframe_deletion	inframe_deletion	30,315
disruptive_inframe_insertion	inframe_insertion	8,424
non_coding_transcript_exon_variant	non_coding_transcript_variant	308,368
5_prime_UTR_premature_start_codon_gain_variant	5_prime_UTR_variant	19,889
splice_acceptor_variant	splicing_variant	42,495
splice_donor_variant	splicing_variant	54,834
conservative_inframe_deletion	inframe_deletion	16,348
stop_retained_variant	synonymous_variant	1,008
conservative_inframe_insertion	inframe_insertion	8,813
start_retained_variant	synonymous_variant	102
3_prime_UTR_truncation&exon_loss_variant	3_prime_UTR_variant	4
5_prime_UTR_truncation&exon_loss_variant	5_prime_UTR_variant	17
ANNOVAR		
upstream	upstream_gene_variant	579,004
intronic	intron_variant	21,439,491
nonsynonymous_SNV	nonsynonymous_variant	417,365
synonymous_SNV	synonymous_variant	704,339
nonframeshift_deletion	inframe_deletion	20,155
nonframeshift_insertion	inframe_insertion	9,809
downstream	downstream_gene_variant	649,889

UTR3	3_prime_UTR_variant	202,677
UTR5	5_prime_UTR_variant	45,245
splicing	splicing_variant	25,407
stopgain	stop_gained	18,004
stoploss	stop_lost	994
exonic	exon_variant	925
intergenic	intergenic_variant	29,197,844
frameshift_insertion	frameshift_variant	23,817
frameshift_deletion	frameshift_variant	151,690
ncRNA_exonic	non_coding_transcript_variant	252,704
ncRNA_intronic	non_coding_transcript_variant	3,410,079
ncRNA_UTR5	non_coding_transcript_variant	7
ncRNA_splicing	non_coding_transcript_variant	1434

Table S4. Precedence Rankings for Annotation Tool Classifications.

Ensembl-VEP	SnpEff	ANNOVAR
transcript_ablation	transcript_ablation	frameshift_insertion
splice_acceptor_variant	chromosome_number_variation	frameshift_deletion
splice_donor_variant	exon_loss_variant	frameshift_block_substitution
stop_gained	frameshift_variant	stopgain
frameshift_variant	stop_gained	stoploss
stop_lost	stop_lost	nonframeshift_insertion
start_lost	start_lost	nonframeshift_deletion
transcript_amplification	splice_acceptor_variant	nonframeshift_block_substitution
feature_elongation	splice_donor_variant	nonsynonymous_SNV
feature_truncation	rare_amino_acid_variant	synonymous_SNV

inframe_insertion	gene_fusion	exonic_unknown
inframe_deletion	bidirectional_gene_fusion	splicing
missense_variant	missense_variant	ncRNA
protein_altering_variant	disruptive_inframe_insertion	UTR5
splice_donor_5th_base_variant	conservative_inframe_insertion	UTR3
splice_region_variant	5_prime_UTR_truncation&exon_loss_variant	intronic
splice_donor_region_variant	3_prime_UTR_truncation&exon_loss_variant	upstream
splice_polypyrimidine_tract_variant	splice_branch_variant	downstream
incomplete_terminal_codon_variant	splice_region_variant	intergenic
start_retained_variant	start_retained_variant	
stop_retained_variant	initiator_codon_variant	
synonymous_variant	synonymous_variant	
coding_sequence_variant	initiator_codon_variant&non_canonical_start_codon	
mature_miRNA_variant	stop_retained_variant	
5_prime_UTR_variant	coding_sequence_variant	
3_prime_UTR_variant	5_prime_UTR_variant	
non_coding_transcript_exon_variant	3_prime_UTR_variant	
intron_variant	5_prime_UTR_premature_start_codon_gain_variant	
NMD_transcript_variant	upstream_gene_variant	
non_coding_transcript_variant	downstream_gene_variant	
coding_transcript_variant	TF_binding_site_variant	
upstream_gene_variant	regulatory_region_variant	
downstream_gene_variant	miRNA	
TFBS_ablation	sequence_feature	
TFBS_amplification	conserved_intron_variant	
TF_binding_site_variant	intron_variant	
regulatory_region_ablation	intragenic_variant	
regulatory_region_amplification	conserved_intergenic_variant	
regulatory_region_variant	intergenic_region	

intergenic_variant	coding_sequence_variant	
sequence_variant	non_coding_transcript_exon_variant	
	non_coding_transcript_variant	
	gene_variant	
	chromosome	
